# When behavior outweighs infrastructure: disinfectant and latex exposure and preventive practice among operating room nurses in China

**DOI:** 10.3389/fpubh.2026.1925091

**Published:** 2026-09-15

**Authors:** PingPing Li, Lixue Zhang, Silan Ren, Jian Song, Xin Li

**Affiliations:** Anzhen Nanchong Hospital, Capital Medical University & Nanchong Central Hospital, The Second Clinical Medical School of North Sichuan Medical University, Nanchong, Sichuan, China

**Keywords:** disinfectants, latex hypersensitivity, occupational exposure, occupational respiratory symptoms, operating-room nurses, preventive practices

## Abstract

**Background:**

Operating-room (OR) nurses encounter disinfectants, sterilizing agents, and latex products as embedded job tasks, yet their multi-domain symptom burden, safety awareness, and preventive practices have rarely been examined together, particularly in Chinese tertiary-care settings.

**Methods:**

We undertook a single-center cross-sectional investigation of operating-room nurses at a tertiary hospital in China. A rigorously structured questionnaire captured agent-specific exposure frequency, task-based exposure, occupational symptomatology, safety awareness, and preventive practices. Composite exposure, awareness, and practice indices were systematically derived. Associations linking exposures, workplace controls, and five binary symptom outcomes were quantified through modified Poisson regression with robust variance estimation, expressed as adjusted prevalence ratios (aPRs).

**Results:**

Among 1,271 OR nurses (87.7% women; mean age 32.9 years), regular (weekly or more) exposure was highest for alcohol-based antiseptics (54.1%), latex products (42.7%), quaternary ammonium compounds (38.6%), and chlorine (32.5%), while high-level sterilants ranked lowest. Work-related mucosal/ocular symptoms were most prevalent (33.2%), followed by skin (22.3%), healthcare or work impact (10.1%), respiratory (8.7%), and latex-compatible symptoms (6.3%). Current smoking (aPR 2.00) and multiple-agent co-exposure (aPR 1.99) were independently associated with a higher prevalence of respiratory symptoms, whereas higher preventive-practice scores were associated with approximately half the prevalence (aPR 0.50). Adequate ventilation was associated with a lower prevalence of mucosal/ocular symptoms (aPR 0.82), and latex exposure was associated with skin symptoms (aPR 1.05 per 10 points). Overall, 644 of 1,271 nurses (50.7%) met the *a priori* threshold for adequate safety awareness (score ≥6/8). Safety infrastructure predicted stronger preventive practice but not lower symptom burden.

**Conclusion:**

Chemical symptom burden among OR nurses was substantial, multi-domain, and behaviorally modifiable. Prevention should combine behavior-specific training, targeted substitution, and ventilation controls with organizational measures addressing workload, supported by active symptom surveillance.

## 1 Introduction

Operating room (OR) nurses occupy one of the most chemically demanding roles in healthcare. Their duties require repeated contact with high-level disinfectants, sterilizing agents, and latex-containing products across every shift—not as incidental encounters, but as embedded job tasks (1–3). Scrub and circulating nurses prepare solutions, soak and rinse instruments, and work in sustained proximity to vapors that other hospital staff rarely encounter. That pattern of task-integrated, shift-long co-exposure sets OR nurses apart from the broader nursing workforce and makes them a group whose occupational health has been systematically understudied (4, 5).

The respiratory consequences of disinfectant exposure in healthcare workers are well documented (3, 6). A systematic review and meta-analysis established that nurses exposed to disinfectants carry a meaningfully higher risk of new-onset asthma than other occupational groups, with bleach and glutaraldehyde identified as the agents most consistently associated with this risk (1, 7). A scoping review of over 130 sources confirmed that occupational asthma develops over years rather than months in this population, and that some formaldehyde and glutaraldehyde air concentrations in active healthcare settings exceed recognized occupational exposure limits (8). In a cross-sectional study at a tertiary hospital, healthcare workers exposed to cleaning agents had significantly higher odds of both respiratory and skin symptoms compared with unexposed colleagues (9). A representative survey of nearly 10,000 health professionals in Texas found that new-onset asthma was independently associated with surface cleaning, use of ortho-phthalaldehyde, bleach and quaternary ammonium compounds, and aerosol spray application (10). A large prospective cohort of US. nurses found no significant association between disinfectant use and incident asthma; the authors attributed this null finding to the healthy worker effect operating in a late career population and explicitly called for study of active clinical workers at earlier career stages (3). More recently, a 2025 analysis of over 5,000 French healthcare workers identified distinct disinfectant and cleaning product exposure clusters, with nurses in the high multi-agent cluster showing significantly elevated asthma symptom burden (11). Alongside these findings, a 2025 cohort study following 75,675 U.S. nurses found that OR nurses specifically had markedly higher cardiovascular disease risk compared with those in non-clinical administrative roles, suggesting that the physiological consequences of OR chemical exposure may extend beyond respiratory and dermatological outcomes (12).

The skin disease burden in this population is substantial and runs parallel to respiratory harm. A large cross-sectional study of hospital employees in Sweden found a one-year hand eczema prevalence of 21%, with soap handwashing and prolonged glove use showing dose-dependent associations (13). Among community care workers in Sweden the prevalence was even higher, exceeding one in three respondents, with occupational water and soap exposure as the principal drivers (14). Quaternary ammonium compounds— among the most frequently used surface disinfectants in OR settings—have been identified as capable of inducing contact dermatitis and sensitization through IgE-mediated mechanisms, a hazard that differs mechanistically from the irritant pathway of bleach and alcohol (15). Natural rubber latex adds a further sensitization dimension; although prevention programs have reduced latex allergy prevalence in high-income countries, the evidence confirms that powdered gloves continue to amplify allergen exposure and that awareness of this risk remains uneven across institutions (1, 16).

The Chinese healthcare context makes these concerns especially pertinent. China's tertiary hospital system operates under chronic workforce pressure, with reported occupational fatigue rates among nurses reaching over 50% in published surveys, and junior staff carrying a disproportionate share of high-intensity task exposure (17). Operating rooms rank consistently among the highest-incidence sites for occupational exposures of multiple types in Chinese hospitals (18). Yet the chemical exposure literature from Chinese OR settings remains thin. Most published work from China addresses needlestick and sharps injuries (18), inhalational anesthetic hazards (19), and nurse role stress (20)—important areas, but ones that leave chemical disinfectant and sterilant exposure largely uncharacterized in this setting. Existing international studies that have examined disinfectant exposure among OR staff tend to measure a single symptom domain, omit formal awareness or preventive practice assessment, or focus on populations whose institutional infrastructure and practice norms differ substantially from Chinese tertiary centers (21–23).

Awareness and practice deficits compound exposure-related risk. A study of South Korean nurses found that both PPE knowledge and awareness were independently associated with compliance, establishing a direct pathway from training to protective behavior (24). Yet training receipt, SDS access, and consistent PPE use remain inconsistently distributed even in well-resourced OR environments, with workload pressure and inadequate equipment fit cited most often as barriers. Therefore, this study was conducted at a single tertiary-care center in China to address these gaps. We aimed to characterize agent-specific occupational exposure frequencies across eleven disinfectant, sterilant, and latex product categories; describe the prevalence of work-related respiratory, skin, mucosal/ocular, and latex-compatible symptoms; assess occupational safety awareness and preventive practice; and identify factors independently associated with symptom outcomes among OR nurses.

## 2 Methods

### 2.1 Study design, setting, and participants

We conducted a cross-sectional study of operating-room (OR) nurses at Capital Medical University & Nanchong Central Hospital, the Second Clinical Medical School of North Sichuan Medical University, Sichuan, 637000, China, between February 2026 and April 2026. All participants were recruited and surveyed during this single three-month data-collection period; no participant data from earlier calendar years were included. Ethics approval was obtained before data collection commenced, and each nurse was surveyed on a single occasion. Over this three-month collection window, the institution's disinfectant and sterilant formulary, personal protective equipment provisioning, and ventilation and local-exhaust systems remained unchanged, and no revision of institutional infection-control or occupational-safety policy occurred. The study characterized occupational exposure to disinfectants, sterilizing agents, cleaning products, and latex-containing materials and examined associations of these exposures with work-related symptoms, occupational safety awareness, and preventive practices. Reporting followed the Strengthening the Reporting of Observational Studies in Epidemiology (STROBE) recommendations for cross-sectional studies (25).

Eligible participants were registered nurses currently employed in OR or perioperative roles who had worked in the OR environment during the study period and were directly involved in surgical assistance, instrument handling, perioperative patient care, environmental disinfection, sterilization-related activities, anesthesia support, or post-anesthesia recovery. Administrative staff without clinical duties, nursing students, interns, temporary observers, and respondents with substantial missing data on key exposure or outcome variables were excluded. As the target population was bounded and enumerable, all eligible OR nurses were invited to participate (census approach), which minimizes sampling bias and maximizes statistical power in single-center occupational studies. The large sample reflects the size of this high-volume tertiary operating-room complex; all perioperative nursing staff across scrub, circulating, recovery/anesthesia-support, and sterilization/instrument-processing roles formed the eligible population. To prevent duplicate participation, each questionnaire was linked to a unique staff identifier, each nurse was surveyed once during the February–April 2026 data-collection period, and responses were screened for duplicate identifiers before analysis. The response rate was calculated as completed questionnaires divided by the number of eligible nurses invited. Of the 2,017 eligible nurses approached, 1,271 completed the questionnaire and met all eligibility criteria, yielding a response rate of 63.0% (1,271/2,017).

### 2.2 Instrument development and data collection

Data were collected with a structured, self-administered questionnaire developed for this study. Items were adapted from validated instruments and prior occupational-health studies addressing healthcare-worker exposure to disinfectants and sterilant, occupational respiratory disease, occupational dermatitis, latex-related symptoms, and workplace safety practice (7, 13, 24). The questionnaire was administered in Mandarin Chinese; source items originally in English were translated into Chinese and independently back-translated by bilingual occupational-health specialists, with discrepancies reconciled by consensus to preserve conceptual equivalence. Content validity was established through review by a panel of three specialists in occupational health, public health, nursing management, and infection prevention, and the instrument was piloted with 32 OR nurses before full deployment, with items refined according to cognitive feedback. Internal consistency of the multi-item scales in the full sample was acceptable (preventive-practice score, Cronbach's α = 0.78; eight-item awareness knowledge score, KR-20 = 0.71.

The final 52-item questionnaire comprised eight domains: sociodemographic and professional characteristics; occupational workload and work organization; exposure to disinfectants, sterilants, cleaning products, antiseptics, and latex materials; exposure-related tasks and accidental incidents; PPE use and workplace controls; occupational safety awareness; preventive practices and barriers; and work-related health symptoms. Items used categorical, ordinal, frequency-based, and continuous response formats as appropriate. Completed questionnaires were screened for completeness and internal consistency before inclusion. Data cleaning comprised range verification, duplicate detection, cross-field logical consistency checks, and coding against predefined operational definitions. Where a screening exposure item conflicted with agent-level responses, agent-level evidence was treated as authoritative, and the screening item recoded accordingly; sensitivity analyses excluding recoded records confirmed robustness of the principal findings.

### 2.3 Sociodemographic, professional, and susceptibility variables

We recorded age, sex, educational attainment, professional designation, primary OR role, total nursing experience, and OR-specific experience. Age was analyzed continuously and in categories (<25, 25–29, 30–34, 35–39, 40–44, ≥45 years). Education was categorized as diploma, bachelor's degree, master's degree or higher, and other. Primary OR role was classified as scrub nurse, circulating nurse, combined scrub/circulating nurse, recovery or anesthesia-support nurse, sterilization or instrument-processing nurse, and other; sparse categories were combined in multivariable models to ensure estimation stability.

Susceptibility factors comprised physician-diagnosed asthma, allergic rhinitis, atopic eczema, family history of asthma or allergic disease, and smoking status (never, former, current). A composite of any atopic history variable was coded positive when a participant reported at least one of asthma, allergic rhinitis, atopic eczema, or family history of allergic disease, and was entered as a potential confounder. Because wet work is an established determinant of occupational skin disease in healthcare workers (13), participants reported average wet-work duration per shift (<1, 1–2, >2–4, >4 h) and frequency of moisturizer or barrier-cream use on a five-point scale from never to always.

### 2.4 Occupational workload

Work-organization variables comprised weekly working hours (<35, 35–44, 45–54, ≥55 h), shifts per week, night-shift frequency (none, 1–3, 4–6, ≥7 per month), and weekly surgical workload (<5, 5–9, 10–14, ≥15 procedures). The highest procedure category was top-coded at 15 for descriptive purposes and analyzed as an ordinal variable, since values above the threshold could not be distinguished. Perceived workload interference with safety adherence and participation in emergency surgery were each assessed on a five-point Likert scale from never to always.

### 2.5 Occupational exposure assessment

Exposure assessment captured agent-specific frequency, task-based exposure, composite exposure indices, accidental incidents, and engineering controls. Participants reported exposure frequency to high-level disinfectants and sterilant [glutaraldehyde, ortho-phthalaldehyde (OPA), formaldehyde or formalin, ethylene oxide, hydrogen peroxide vapor or plasma, peracetic acid], environmental and antiseptic agents [chlorine or hypochlorite, quaternary ammonium compounds (QACs), alcohol-based disinfectants, chlorhexidine, povidone-iodine], and latex sources (latex gloves and products, powdered latex gloves). These agents were selected because they are recognized as respiratory and dermal sensitizers in healthcare settings (7, 11). Frequency was recorded in six categories: never, less than once per month, monthly, weekly, daily or almost daily, and not sure. For analysis, exposures were dichotomized into regular (weekly or more) versus infrequent or no exposure.

Task-based exposure covered preparation, dilution, or pouring of disinfectants; manual cleaning or soaking of instruments; surface disinfection by spraying or wiping; and handling of chemically processed equipment, using the same frequency categories. Accidental chemical splashes, spill, or skin-contact incidents were recorded as none, one, two to three, or four or more, and dichotomized as any versus none for regression. A composite disinfectant and sterilant exposure score was derived from the nine non-latex agent items reported in Table 1 (six high-level disinfectants/sterilant—glutaraldehyde, ortho-phthalaldehyde, formaldehyde, ethylene oxide, hydrogen peroxide, and peracetic acid—and three environmental/antiseptic groups—chlorine/hypochlorite, quaternary ammonium compounds, and the combined alcohol/chlorhexidine/povidone-iodine group). Each frequency was assigned an ordinal value from 0 (never) to 4 (daily or almost daily), with “not sure” coded as 0 on the conservative assumption that a respondent unable to recall an agent was unlikely to have frequent, salient exposure to it; “not sure” was additionally examined as missing in sensitivity analyses, which did not materially change the results. The summed score was standardized to a 0–100 scale as (observed score ÷ maximum possible score) × 100, with higher values indicating greater cumulative exposure. A latex exposure score (0–100) was derived identically from the two latex-source items (latex gloves/products and powdered latex gloves). A binary multiple high-exposure cluster variable identified nurses reporting concurrent regular exposure to several disinfectant, cleaning-product, or sterilant categories, reflecting the heterogeneous multi-agent co-exposure pattern characteristic of perioperative work (11, 16).

**Table 1:** Agent–specific exposure frequencies and unadjusted association with work–related respiratory symptoms.

Agent/product group	Never	<1/ month	Monthly	Weekly	Daily/almost daily	Not sure	Weekly or more	Respiratory symptoms among weekly–or–more exposed	Unadjusted PR (95% CI)	*p* value
Glutaraldehyde	424 (33.4)	241 (19.0)	248 (19.5)	196 (15.4)	104 (8.2)	58 (4.6)	300 (23.6)	35/300 (11.7)	1.49 (1.02–2.18)	0.039
Ortho–phthalaldehyde	487 (38.3)	273 (21.5)	177 (13.9)	159 (12.5)	51 (4.0)	124 (9.8)	210 (16.5)	19/210 (9.0)	1.04 (0.65–1.67)	0.86
Formalin/formaldehyde	446 (35.1)	299 (23.5)	218 (17.2)	188 (14.8)	77 (6.1)	43 (3.4)	265 (20.8)	32/265 (12.1)	1.54 (1.04–2.27)	0.03
Ethylene oxide	522 (41.1)	290 (22.8)	159 (12.5)	110 (8.7)	26 (2.0)	164 (12.9)	136 (10.7)	12/136 (8.8)	1.01 (0.57–1.79)	0.969
Hydrogen peroxide vapor/plasma	442 (34.8)	300 (23.6)	190 (14.9)	154 (12.1)	58 (4.6)	127 (10.0)	212 (16.7)	22/212 (10.4)	1.23 (0.79–1.92)	0.353
Peracetic acid	570 (44.8)	252 (19.8)	161 (12.7)	105 (8.3)	18 (1.4)	165 (13.0)	123 (9.7)	13/123 (10.6)	1.24 (0.72–2.14)	0.448
Chlorine/hypochlorite	345 (27.1)	214 (16.8)	247 (19.4)	217 (17.1)	196 (15.4)	52 (4.1)	413 (32.5)	44/413 (10.7)	1.36 (0.95–1.96)	0.092
QACs/surface sprays or wipes	269 (21.2)	185 (14.6)	222 (17.5)	253 (19.9)	237 (18.6)	105 (8.3)	490 (38.6)	42/490 (8.6)	0.97 (0.67–1.40)	0.871
Alcohol/CHG/PVP–iodine	195 (15.3)	159 (12.5)	189 (14.9)	280 (22.0)	407 (32.0)	41 (3.2)	687 (54.1)	58/687 (8.4)	0.93 (0.65–1.33)	0.69
Latex gloves/products	264 (20.8)	186 (14.6)	220 (17.3)	255 (20.1)	288 (22.7)	58 (4.6)	543 (42.7)	49/543 (9.0)	1.06 (0.74–1.52)	0.751
Powdered latex gloves	517 (40.7)	287 (22.6)	180 (14.2)	145 (11.4)	48 (3.8)	94 (7.4)	193 (15.2)	15/193 (7.8)	0.87 (0.52–1.47)	0.608

p–values compare weekly–or–more exposure with less–than–weekly/no/not-sure exposure for the primary outcome, work-related respiratory symptoms. PR, prevalence ratio; QACs, quaternary ammonium compounds; CHG, chlorhexidine gluconate; PVP-iodine, povidone-iodine.

### 2.6 Workplace controls, PPE, and preventive practices

Participants reported the availability and functioning of ventilation or local exhaust controls in areas where disinfectants and sterilant were used (always available and functioning; available but inconsistently functioning; available but rarely used; not available; not sure); adequate ventilation was defined as systems consistently available and functioning. PPE use during chemical handling was assessed for chemical-resistant gloves, eye protection or face shields, and protective gowns or aprons on a five-point frequency scale, with consistent use defined as “often” or “always.”

Six preventive behaviors were assessed: using chemical-appropriate gloves; using eye protection during splash-risk tasks; keeping containers closed when not in use; avoiding unnecessary spraying; reviewing labels, safety data sheets (SDS), or instructions; and reporting spills, splash incidents, or symptoms. Each was scored 0 (never) to 4 (always), with “not applicable” treated as missing for that item; a preventive-practice score was computed as the mean across items when at least four of six were answered, ranging from 0 to 4. The principal barrier to consistent protection was recorded from a fixed list. Participants also reported availability of latex-free alternatives, SDS and written standard operating procedures, and receipt of formal occupational safety training in the previous 12 months (any versus none). These resource and training measures were included because awareness and training have been shown to predict PPE compliance among nurses (24).

### 2.7 Occupational safety awareness

Knowledge was assessed with eight true/false/do-not-know statements addressing the irritant and respiratory hazards of high-level disinfectants and sterilants; the asthma-related effects of glutaraldehyde, formaldehyde, and OPA; appropriate chemical-resistant glove selection; the protective limitations of latex gloves; safe container storage; spill-response adequacy; the informational content of SDS; and powdered-latex allergen exposure. Correct responses scored 1; incorrect and “do not know” responses scored 0. The total awareness score ranged from 0 to 8, and adequate awareness was defined *a priori* as 6 or higher. Knowledge of the institutional reporting pathway was recorded separately.

### 2.8 Outcome measures

The primary outcome was work-related respiratory symptoms, defined as cough, wheeze, chest tightness, or shortness of breath occurring during or after OR duties at least “sometimes,” with symptom improvement away from work used to establish work-relatedness, consistent with established occupational-asthma criteria. Five secondary outcomes were defined. Work-related skin symptoms comprised hand or forearm itching, redness, dryness, rash, fissuring, or dermatitis-like changes occurring during occupational activity. Work-related mucosal or ocular symptoms comprised nasal irritation, rhinorrhea, sneezing, sore throat, eye irritation, tearing, or conjunctival discomfort. Latex-compatible symptoms comprised immediate itching, urticaria, nasal or ocular symptoms, or respiratory discomfort temporally associated with latex exposure; this represented symptom-based surveillance and was not interpreted as confirmed latex allergy (16). Healthcare utilization or work impact was coded positive when symptoms led to medical consultation, medication use, sick leave, modified duties, or a transfer request. All outcomes were self-reported and intended for epidemiological assessment; none was interpreted as a clinical diagnosis of asthma, occupational asthma, chronic obstructive pulmonary disease, contact dermatitis, or latex allergy.

### 2.9 Statistical analysis

Continuous variables were summarized as means with standard deviations or medians with interquartile ranges according to distribution, assessed by inspection and the Shapiro-Wilk test; categorical variables were summarized as frequencies and percentages. Prevalence estimates for the derived outcomes were reported with Wilson 95% confidence intervals (CIs), which perform well for binomial proportions across the prevalence range. Bivariable associations were examined using the chi-square test, Fisher's exact test where expected cell counts were small, and independent-sample *t*-tests or Mann-Whitney U tests for continuous comparisons. Agent-specific associations with the primary outcome were summarized as crude prevalence ratios (PRs) with 95% CIs, contrasting regular (weekly or more) with less-than-regular exposure.

Because all outcomes were common and binary, adjusted associations were estimated using modified Poisson regression with robust (sandwich) variance estimation, which yields directly interpretable prevalence ratios and avoids the overestimation that affects odds ratios when outcomes are frequent (8, 26). Separate multivariable models were fitted for each of the five binary outcomes. Covariates were selected as *a priori* on the basis of biological plausibility and prior occupational-health evidence rather than by stepwise procedures, and all pre-specified covariates were entered simultaneously. Each model included 20 parameters on 1,271 complete cases, giving approximately 5–6 events per variable for the primary respiratory outcome (111 events); events per variable were lower for the rarer outcomes (latex-compatible, 80; healthcare/work impact, 129), whose estimates should be interpreted conservatively. Collinearity was low (all generalized variance-inflation factors <2.5), and a parsimonious model restricted to the *a priori* primary predictors (smoking, high-exposure cluster, exposure scores, ventilation, and preventive-practice score) yielded materially unchanged estimates for the primary outcome. Models adjusted for age, sex, educational level, OR role, OR experience, weekly working hours, smoking status, any atopic history, wet-work exposure, composite disinfectant and sterilant exposure score, latex exposure score, accidental exposure incidents, ventilation adequacy, occupational safety training, preventive-practice score, and multiple high-exposure cluster status. Adjusted prevalence ratios (aPRs) with 95% CIs were reported, with continuous exposure scores modeled per 10-point increment. A total of 48 submitted questionnaires were excluded before analysis because of substantial missing data on key exposure or outcome variables, leaving 1,271 records. Among these analyzed records, item-level missingness on key exposure, awareness, preventive-practice, and outcome variables was low (below 2% for any single item). Analyses used complete-case methods after exclusion of records missing key exposure or outcome data. A sensitivity analysis restricted to participants with fully complete key variables did not materially change the principal findings. All tests were two-sided with significance set at *p* < 0.05. Because five outcomes were examined, the potential for type I error inflation was acknowledged and addressed by pre-specifying a single primary outcome. The Safety Infrastructure Index (SII, range 0–4) summed four binary institutional-provision indicators, each scored 1 when present: adequate ventilation or local exhaust, availability of latex-free alternatives, availability of safety data sheets and written standard operating procedures, and formal occupational safety training in the previous 12 months. Internal consistency was assessed with Cronbach's α, between-role differences with Kruskal–Wallis, and determinants of higher SII with ordered logistic regression. A path model then tested infrastructure's association with symptom burden through behavior, specifying the SII as exogenous variable, the preventive-practice score as mediator, and total symptom burden (count of affected domains, 0–5) as outcome, adjusting for the same *a priori* covariates; standardized coefficients were estimated and the indirect effect tested by bias-corrected bootstrap (500 replications, 95% CI). All statistical analysis were performed in R (version 4.3.2; R Foundation for Statistical Computing, Vienna, Austria).

## 3 Results

### 3.1 Participant characteristics

Of 1,271 operating-room (OR) nurses analyzed, the mean age was 32.9 ± 7.2 years and 1,115 (87.7%) were women (Table 2). Most held a bachelor's degree (76.3%), and the largest role group performed combined scrub and circulating duties (38.9%), followed by scrub nurses (26.3%) and circulating nurses (23.5%). Median OR-specific experience was 4.0 years (IQR 2.0–8.0). Susceptibility factors were common: allergic rhinitis was reported by 23.3%, a family history of allergy or asthma by 21.6%, atopic eczema by 15.3%, and prior asthma by 7.9%. Prolonged wet work exceeding 2 h per shift was reported by 65.3% of participants, while current smoking was infrequent (5.3%). This profile—a young, predominantly female, degree-qualified workforce with a substantial atopic and wet-work background—defines a population whose chemical exposure has rarely been characterized in detail in the Chinese perioperative setting, and against which the following exposure and symptom findings should be read.

**Table 2:** Participant characteristics and susceptibility covariates.

Characteristic	Total sample (*N* = 1,271)
Age, years, mean ± SD	32.93 ± 7.24
Age, years, median (IQR)	32.0 (27.0–37.0)
**Age group**	
<25 years	95 (7.5)
25–29 years	371 (29.2)
30–34 years	364 (28.6)
35–39 years	235 (18.5)
40–44 years	126 (9.9)
≥45 years	80 (6.3)
**Sex**	
Female	1,115 (87.7)
Male	144 (11.3)
Prefer not to say	12 (0.9)
**Highest nursing qualification**	
Diploma in nursing	212 (16.7)
Bachelor's degree	970 (76.3)
Master's degree or above	76 (6.0)
Other	13 (1.0)
**Professional designation**	
Junior staff nurse	245 (19.3)
Staff nurse	598 (47.0)
Senior staff nurse	332 (26.1)
Charge nurse/head nurse	79 (6.2)
Other	17 (1.3)
**Primary operating–room role**	
Scrub nurse	334 (26.3)
Circulating nurse	299 (23.5)
Both scrub and circulating nurse	495 (38.9)
Recovery/anesthesia support nurse	99 (7.8)
Sterilization or instrument–processing–related role	39 (3.1)
Other	5 (0.4)
Total nursing experience, years, mean ± SD	7.66 ± 5.82
Total nursing experience, years, median (IQR)	6.0 (3.0–11.0)
**Total nursing experience category**	
<1 year	68 (5.4)
1–4 years	397 (31.2)
5–9 years	384 (30.2)
10–14 years	258 (20.3)
≥15 years	164 (12.9)
OR–specific experience, years, mean ± SD	5.06 ± 4.38
OR–specific experience, years, median (IQR)	4.0 (2.0–8.0)
<1 year	161 (12.7)
1–4 years	512 (40.3)
5–9 years	412 (32.4)
10–14 years	134 (10.5)
≥15 years	52 (4.1)
**Prior asthma**	
No	1,170 (92.1)
Yes	101 (7.9)
**Allergic rhinitis**	
No	975 (76.7)
Yes	296 (23.3)
**Atopic eczema**	
No	1,077 (84.7)
Yes	194 (15.3)
**Family history of allergy/asthma**	
No	997 (78.4)
Yes	274 (21.6)
**Smoking status**	
Never smoker	1,151 (90.6)
Former smoker	53 (4.2)
Current smoker	67 (5.3)
**Wet–work covariates**	
<1 h/shift	96 (7.6)
1–2 h/shift	346 (27.2)
>2–4 h/shift	583 (45.9)
>4 h/shift	246 (19.4)
**Moisturizer/barrier–cream use**	
Never	155 (12.2)
Rarely	257 (20.2)
Sometimes	436 (34.3)
Often	304 (23.9)
Always	119 (9.4)

Values are *n* (%) unless otherwise stated. Baseline p–values are not shown because this table describes the analytic sample rather than exposure–outcome associations. OR, operating room; IQR, interquartile range.

### 3.2 Workload and occupational exposure

Participants worked a mean of 47.1 ± 8.5 h weekly, and 25.1% reported that time pressure “always” compromised safety compliance (Table 3). The composite disinfectant and sterilant exposure score averaged 33.9 ± 11.2 (0–100 scale), and 10.5% of nurses fell into the multiple high-exposure cluster. Agent-level exposure was dominated by antiseptics and surface disinfectants (Figure 1A). Regular (weekly or more) exposure was most frequent for alcohol/chlorhexidine/povidone-iodine (54.1%), latex products (42.7%), quaternary ammonium compounds (38.6%), and chlorine/hypochlorite (32.5%); high-level sterilants such as ethylene oxide (10.7%) and peracetic acid (9.7%) ranked lowest (Figure 1B, Table 1). This gradient is itself a key finding: it shows that the dominant exposure burden in modern ORs arises from frequent, low-perceived-risk antiseptics and surface agents rather than from the classic high-level sterilants that have historically attracted regulatory attention. The exposure-control balance was unfavorable: although appropriate glove use (78.5%) and formal training (63.7%) were relatively common, adequate ventilation reached only 45.2% and consistent eye protection only 32.3% (Figure 1C), revealing that protective effort concentrates on the hand while airway and ocular protection lag. Outcome prevalence rose steadily across exposure-score quintiles for skin symptoms; respiratory symptom prevalence fluctuated across the lower quintiles before rising sharply in the top quintile (Figure 1D).

**Table 3:** Workload, exposure intensity, task–based exposure, and environmental controls.

Characteristic	Total sample (*N* = 1,271)
Weekly working hours, mean ± SD	47.06 ± 8.45
Weekly working hours, median (IQR)	47.0 (40.0–53.0)
**Weekly working hours**	
<35 h	59 (4.6)
35–44 h	435 (34.2)
45–54 h	560 (44.1)
≥55 h	217 (17.1)
Shifts per week, mean ± SD	4.76 ± 1.37
**Shifts per week**	
1–2 shifts	58 (4.6)
3–4 shifts	374 (29.4)
5 shifts	530 (41.7)
≥6 shifts	309 (24.3)
Night shifts/month, mean ± SD	3.25 ± 2.62
**Night shifts/month**	
No night shifts	217 (17.1)
1–3	530 (41.7)
4–6	384 (30.2)
≥7	140 (11.0)
Surgical procedures/week, mean ± SD	10.59 ± 3.89
**Surgical procedures/week**	
<5	91 (7.2)
5–9	335 (26.4)
10–14	576 (45.3)
≥15	269 (21.2)
**Emergency surgery participation**	
Never	72 (5.7)
Rarely	178 (14.0)
Sometimes	407 (32.0)
Often	438 (34.5)
Always	176 (13.8)
**Time pressure affects safety compliance**	
Never	41 (3.2)
Rarely	138 (10.9)
Sometimes	382 (30.1)
Often	391 (30.8)
Always	319 (25.1)
Disinfectant/sterilant exposure score, mean ± SD (95% CI)	33.91 ± 11.24 (33.29–34.53)
Disinfectant/sterilant exposure score, median (IQR)	33.3 (25.0–41.7)
Latex exposure score, mean ± SD (95% CI)	37.54 ± 23.81 (36.23–38.85)
Latex exposure score, median (IQR)	37.5 (25.0–50.0)
Multiple high DCP exposure cluster	134 (10.5)
Never	409 (32.2)
Less than once per month	264 (20.8)
Monthly	230 (18.1)
Weekly	233 (18.3)
Daily or almost daily	116 (9.1)
Not sure	19 (1.5)
**Task–based exposure: manual cleaning/soaking instruments**	
Never	425 (33.4)
Less than once per month	256 (20.1)
Monthly	218 (17.2)
Weekly	212 (16.7)
Daily or almost daily	130 (10.2)
Not sure	30 (2.4)
**Task–based exposure: surface disinfection by spraying/wiping**	
Never	231 (18.2)
Less than once per month	154 (12.1)
Monthly	206 (16.2)
Weekly	270 (21.2)
Daily or almost daily	390 (30.7)
Not sure	20 (1.6)
**Accidental splash/spill/skin contact**	
No	1,120 (88.1)
Yes, once	73 (5.7)
Yes, 2–3 times	59 (4.6)
Yes, ≥4 times	19 (1.5)
**Ventilation/fume extraction**	
Yes, always available and functioning	575 (45.2)
Available but not always functioning	329 (25.9)
Available but rarely used	152 (12.0)
Not available	86 (6.8)
Not sure	129 (10.1)

Exposure scores are scaled from 0 to 100; higher scores indicate more frequent exposure. DCP, disinfectants, cleaning products, and sterilizing/sterilant products.

**Figure 1:**
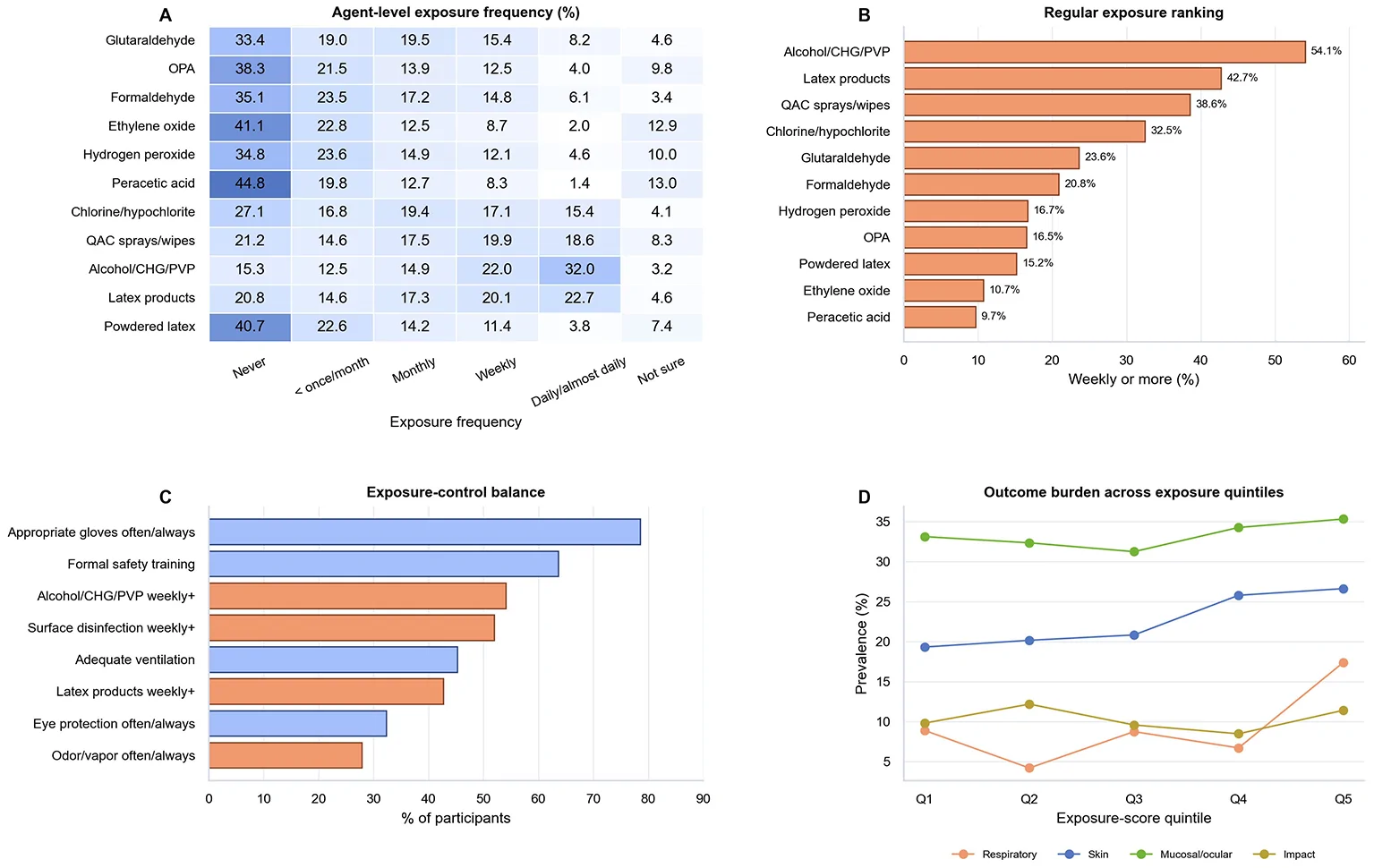
Occupational exposure landscape and exposure-control balance. **(A)** Agent-level exposure frequency for 11 disinfectant, sterilant, and latex product categories, shown as the percentage of nurses reporting each frequency (never, less than once monthly, monthly, weekly, daily or almost daily, not sure). **(B)** Ranking of agents by regular (weekly or more) exposure, expressed as the percentage of participants. **(C)** Exposure-control balance, comparing the prevalence of protective measures (blue) and regular exposures (orange) across the cohort. **(D)** Prevalence of each work-related symptom outcome across quintiles (Q1–Q5) of the composite disinfectant/sterilant exposure score. Exposure scores were scaled 0–100, with higher values indicating more frequent exposure. *N* = 1,271.

### 3.3 Symptom burden and co-occurrence

Work-related mucosal or ocular symptoms were the most prevalent outcome (33.2%, 95% CI 30.7–35.8), followed by skin symptoms (22.3%, 20.1–24.6), healthcare or work impact (10.1%, 8.6–11.9), respiratory symptoms (8.7%, 7.3–10.4), and latex-compatible symptoms (6.3%, 5.1–7.8) (Figure 2A, Table 4). This multi-domain quantification directly addresses a gap in prior work, most of which has measured respiratory or skin outcomes in isolation; by capturing all four symptom domains in a single cohort, the data reveal that the dominant burden in OR nurses is mucosal/ocular rather than respiratory—the very domain least often examined. Isolated mucosal/ocular symptoms (20.8%) and isolated skin symptoms (11.8%) were the commonest single presentations, while the skin–mucosal/ocular combination (4.5%) was the most frequent multi-domain overlap (Figure 2B). Distributions of DCP exposure, awareness, and preventive-practice scores stratified by respiratory-symptom status are shown in Figure 2C. Among nurses reporting a healthcare or work impact, medical consultation alone (3.9%) was the predominant consequence, though sick leave (1.6%) and work modification or transfer requests (1.0%) were also reported (Figure 2D), demonstrating that symptom burden in this population already translates into measurable workforce and service costs.

**Figure 2:**
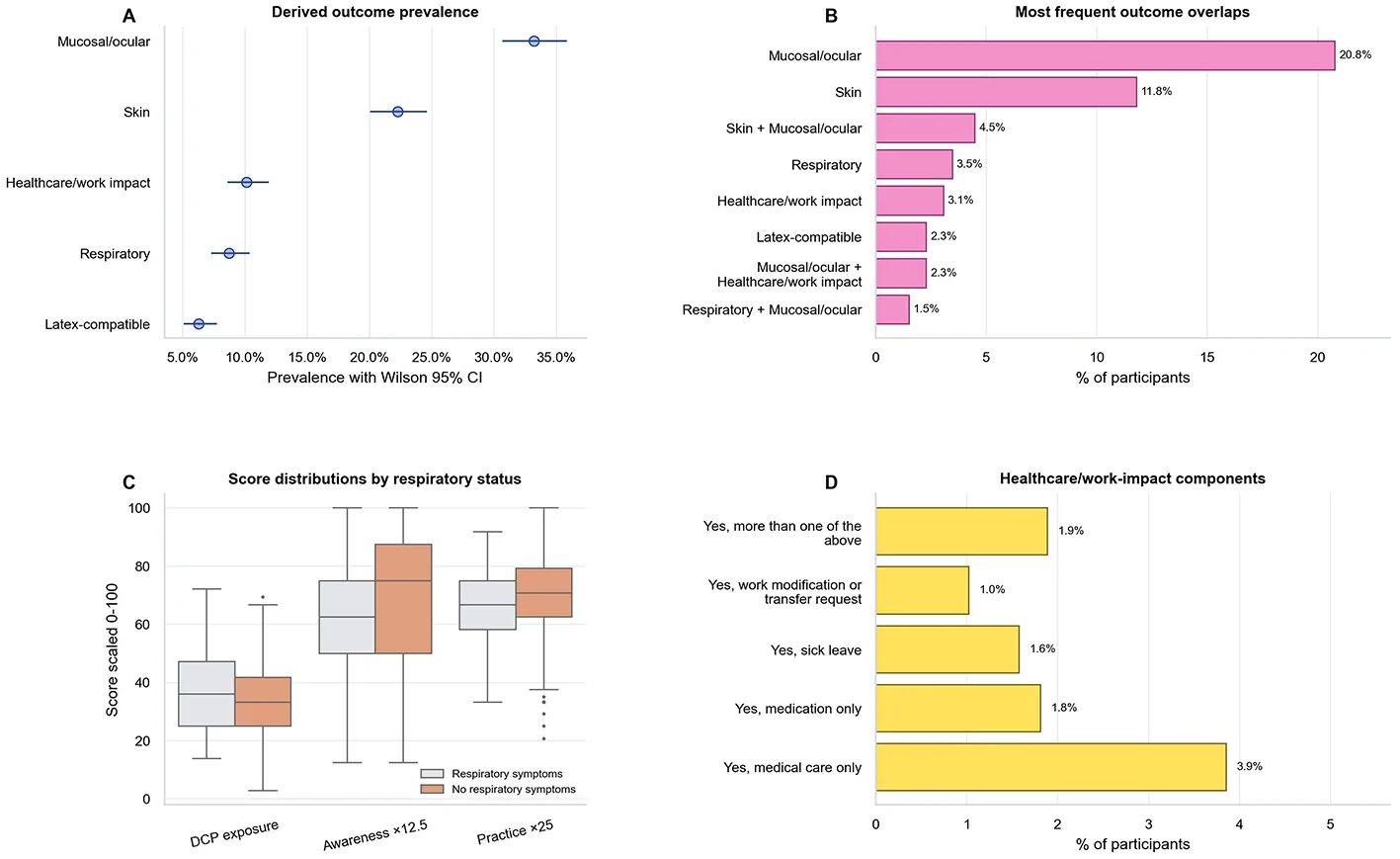
Symptom burden, co-occurrence, and work impact. **(A)** Prevalence of the five derived symptom outcomes with Wilson 95% confidence intervals. **(B)** Most frequent single-domain and multi-domain symptom combinations, expressed as the percentage of participants. **(C)** Distributions of the disinfectant/cleaning-product (DCP) exposure, awareness, and preventive-practice scores (all scaled 0–100) stratified by respiratory-symptom status; boxes show median and interquartile range, whiskers the 1.5 × IQR range, and points the outliers. **(D)** Components of the healthcare or work-impact outcome, expressed as the percentage of participants reporting each consequence. *N* = 1,271.

**Table 4:** Adjusted associations between occupational exposures, workplace controls, and work-related symptom outcomes.

Predictor	Respiratory symptoms aPR (95% CI); *p*	Skin symptoms aPR (95% CI); *p*	Mucosal/ocular symptoms aPR (95% CI); *p*	Latex–compatible symptoms aPR (95% CI); *p*	Healthcare/work impact aPR (95% CI); *p*
Qualification: diploma/other vs bachelor's	0.74 (0.44–1.25); *p* = 0.256	1.11 (0.86–1.44); *p* = 0.416	0.97 (0.79–1.20); *p* = 0.787	1.70 (1.05–2.75); *p* = 0.031	1.54 (1.06–2.24); *p* = 0.024
Qualification: master's or above vs bachelor's	1.18 (0.56–2.47); *p* = 0.666	0.90 (0.57–1.45); *p* = 0.673	1.32 (1.01–1.73); *p* = 0.042	1.39 (0.60–3.23); *p* = 0.439	0.68 (0.28–1.62); *p* = 0.383
OR role: circulating vs both scrub/circulating	0.76 (0.46–1.27); *p* = 0.304	1.19 (0.91–1.56); *p* = 0.198	0.91 (0.74–1.13); *p* = 0.409	0.89 (0.47–1.72); *p* = 0.738	0.77 (0.50–1.19); *p* = 0.241
OR role: scrub vs both scrub/circulating	1.18 (0.80–1.74); *p* = 0.403	1.11 (0.85–1.44); *p* = 0.445	1.04 (0.85–1.26); *p* = 0.716	1.45 (0.86–2.47); *p* = 0.167	0.79 (0.52–1.21); *p* = 0.283
OR role: support/sterilization/other vs both scrub/circulating	0.47 (0.20–1.07); *p* = 0.072	1.16 (0.82–1.63); *p* = 0.393	1.11 (0.87–1.43); *p* = 0.389	1.75 (0.91–3.35); *p* = 0.093	0.99 (0.58–1.66); *p* = 0.955
Age, per 5–year increment	1.00 (0.78–1.27); *p* = 0.987	1.10 (0.95–1.28); *p* = 0.212	1.07 (0.96–1.21); *p* = 0.221	0.94 (0.69–1.26); *p* = 0.666	0.87 (0.70–1.08); *p* = 0.212
Female sex	1.72 (0.87–3.38); *p* = 0.117	0.98 (0.72–1.34); *p* = 0.913	0.87 (0.69–1.10); *p* = 0.246	0.92 (0.47–1.80); *p* = 0.806	0.76 (0.48–1.22); *p* = 0.262
OR–specific experience, per 5 years	0.92 (0.62–1.38); *p* = 0.702	0.97 (0.76–1.24); *p* = 0.818	0.91 (0.75–1.09); *p* = 0.306	1.07 (0.64–1.79); *p* = 0.794	1.15 (0.78–1.69); *p* = 0.475
Weekly working hours, per 10 h	1.02 (0.84–1.26); *p* = 0.814	1.05 (0.94–1.19); *p* = 0.387	0.97 (0.88–1.06); *p* = 0.468	0.98 (0.77–1.25); *p* = 0.858	0.94 (0.79–1.13); *p* = 0.539
Any atopic history	1.29 (0.90–1.83); *p* = 0.165	1.08 (0.88–1.33); *p* = 0.436	1.05 (0.89–1.22); *p* = 0.574	1.35 (0.87–2.08); *p* = 0.182	0.99 (0.72–1.37); *p* = 0.975
Current smoker	2.00 (1.19–3.35); *p* = 0.009	1.16 (0.76–1.76); *p* = 0.491	0.88 (0.60–1.28); *p* = 0.511	1.14 (0.42–3.05); *p* = 0.800	0.51 (0.19–1.39); *p* = 0.187
Former smoker	1.30 (0.54–3.14); *p* = 0.558	0.91 (0.53–1.58); *p* = 0.744	0.43 (0.23–0.81); *p* = 0.010	0.97 (0.31–3.08); *p* = 0.964	0.54 (0.19–1.53); *p* = 0.250
Disinfectant/sterilant exposure score, per 10 points	1.09 (0.88–1.35); *p* = 0.442	1.13 (1.00–1.27); *p* = 0.054	1.01 (0.93–1.11); *p* = 0.778	1.09 (0.84–1.42); *p* = 0.522	0.96 (0.80–1.16); *p* = 0.681
Latex exposure score, per 10 points	0.99 (0.93–1.06); *p* = 0.827	1.05 (1.01–1.10); *p* = 0.014	0.99 (0.96–1.02); *p* = 0.431	1.01 (0.92–1.11); *p* = 0.761	0.99 (0.93–1.06); *p* = 0.782
Accidental splash/spill/skin contact	1.00 (0.57–1.74); *p* = 0.991	1.20 (0.89–1.62); *p* = 0.222	0.86 (0.67–1.12); *p* = 0.266	0.96 (0.48–1.92); *p* = 0.910	1.25 (0.80–1.97); *p* = 0.332
Adequate ventilation/fume extraction	0.88 (0.62–1.26); *p* = 0.490	0.99 (0.81–1.22); *p* = 0.952	0.82 (0.70–0.96); *p* = 0.016	1.26 (0.83–1.91); *p* = 0.288	0.85 (0.61–1.18); *p* = 0.330
Formal safety training in past 12 months	1.24 (0.81–1.90); *p* = 0.314	1.02 (0.80–1.31); *p* = 0.864	0.99 (0.82–1.19); *p* = 0.881	0.82 (0.48–1.39); *p* = 0.457	1.04 (0.71–1.51); *p* = 0.848
Preventive–practice score, per 1 point	0.50 (0.35–0.71); *p* = <0.001	1.08 (0.86–1.35); *p* = 0.522	0.96 (0.81–1.14); *p* = 0.641	1.37 (0.83–2.27); *p* = 0.220	0.92 (0.64–1.32); *p* = 0.652
Multiple high DCP exposure cluster	1.99 (1.06–3.73); *p* = 0.033	1.00 (0.68–1.47); *p* = 0.986	1.01 (0.72–1.41); *p* = 0.952	0.88 (0.36–2.14); *p* = 0.775	1.22 (0.79–1.88); *p* = 0.372

aPRs were estimated using modified Poisson regression with robust standard errors. The model adjusted simultaneously for all variables shown. Reference categories: male/prefer not to say for female sex; bachelor's degree for qualification; both scrub and circulating nurse for OR role; never smoker for smoking. Sparse categories were combined for modeling stability as specified in the methods.

### 3.4 Exposure clustering and cumulative burden

K-means clustering identified three exposure phenotypes (Figure 3A). The high co-exposure cluster (*n* = 272) carried the greatest respiratory burden (12.1%), exceeding the intermediate mixed-exposure (7.5%) and lower-exposure (8.2%) clusters. This phenotype-level signal—that simultaneous multi-agent exposure, rather than any single chemical, marks the highest-risk group—provides empirical support for treating co-exposure as the relevant unit of risk, a concept seldom operationalized in single-center OR studies. Subgroup analysis showed the highest mucosal/ocular vulnerability among support and sterilization staff (36.4%) and scrub/junior nurses (36.0%), and the highest skin-symptom burden among senior circulating (27.5%) and senior scrub (25.7%) nurses (Figure 3B), identifying specific role–seniority strata for targeted surveillance. The distribution of cumulative symptom domains followed a Poisson pattern (AIC 2,917.3), with most nurses affected in zero or one domain and a minority experiencing multi-domain burden (Figure 3C). Inter-variable correlations were generally weak, the notable exception being the moderate awareness–practice correlation (ρ = 0.55) (Figure 3D), indicating that exposure and symptom domains operate largely independently and that knowledge and behavior, rather than exposure intensity alone, move together.

**Figure 3:**
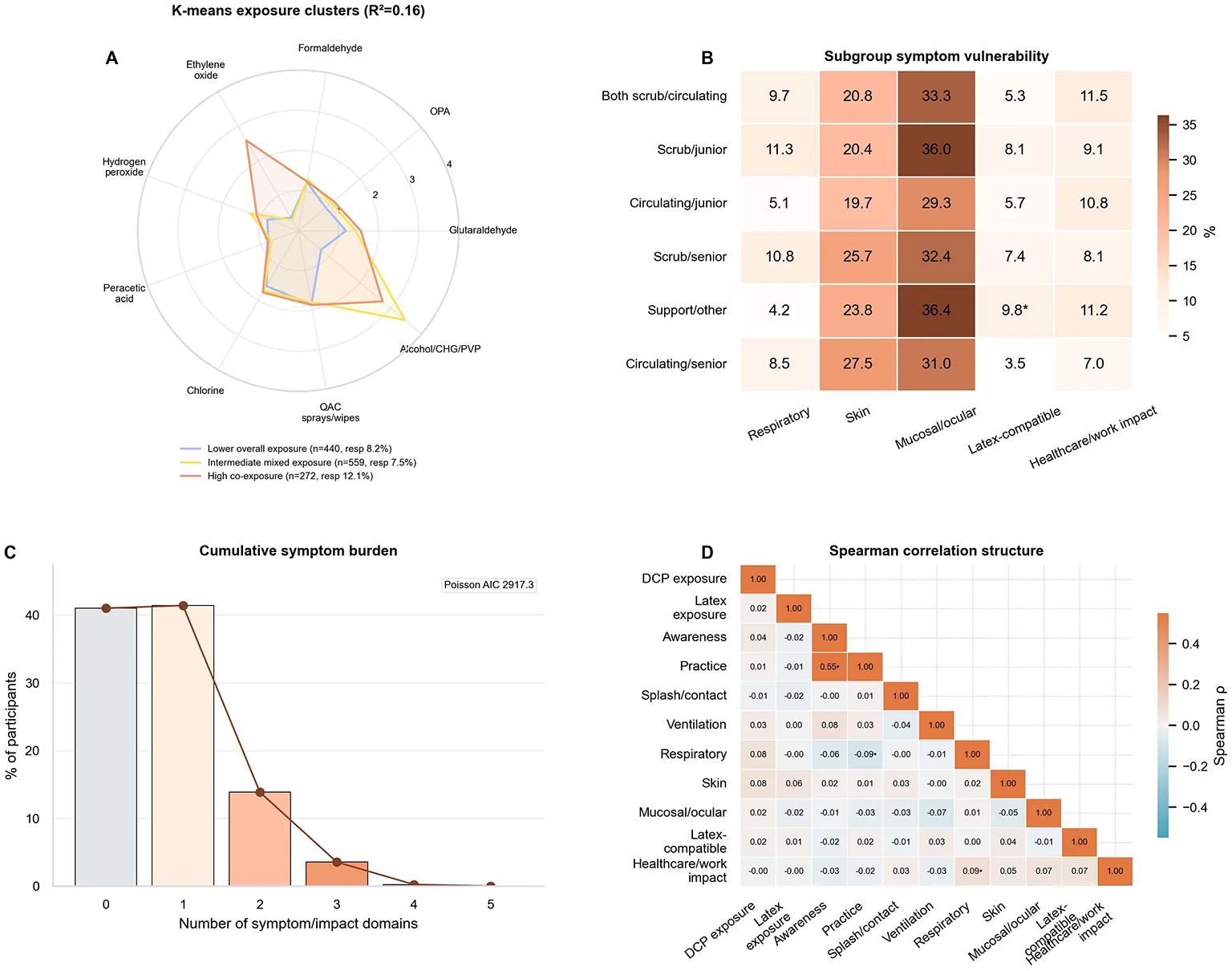
Occupational risk profiling and cumulative symptom burden. **(A)** K-means clustering of nurses by agent-level exposure, identifying three exposure phenotypes (lower overall, intermediate mixed, and high co-exposure); the radar axes show mean exposure frequency per agent, and the cluster legend reports size and respiratory-symptom prevalence. **(B)** Subgroup symptom-vulnerability heatmap showing the prevalence (%) of each outcome across OR role and seniority strata; asterisks denote subgroups exceeding the overall prevalence. **(C)** Distribution of the cumulative number of affected symptom or impact domains per nurse, with the fitted Poisson model (AIC reported). **(D)** Spearman correlation matrix of exposure, awareness, practice, control, and outcome variables; dots indicate statistically significant correlations. *N* = 1,271.

### 3.5 Adjusted associations

In modified Poisson models adjusting for all covariates simultaneously, three predictors were independently associated with work-related respiratory symptoms (Figure 4A, Table 4). Current smoking was associated with a twofold higher prevalence (aPR 2.00, 95% CI 1.19–3.35; *p* = 0.009), and membership in the multiple high DCP exposure cluster was associated with a nearly twofold higher prevalence (aPR 1.99, 95% CI 1.06–3.73; *p* = 0.033). Conversely, each one-point increase in the preventive-practice score was associated with approximately half the prevalence of respiratory symptoms (aPR 0.50, 95% CI 0.35–0.71; *p* < 0.001), the strongest protective association in the analysis. Because these estimates derive from a single cross-sectional assessment, they should be interpreted as associations rather than causal effects; nevertheless, the coexistence of an elevated prevalence ratio associated with multi-agent co-exposure and a markedly lower prevalence ratio associated with preventive practice is the central actionable finding, suggesting that the respiratory symptom burden, although exposure-related, may be associated with individually modifiable behavior.

**Figure 4:**
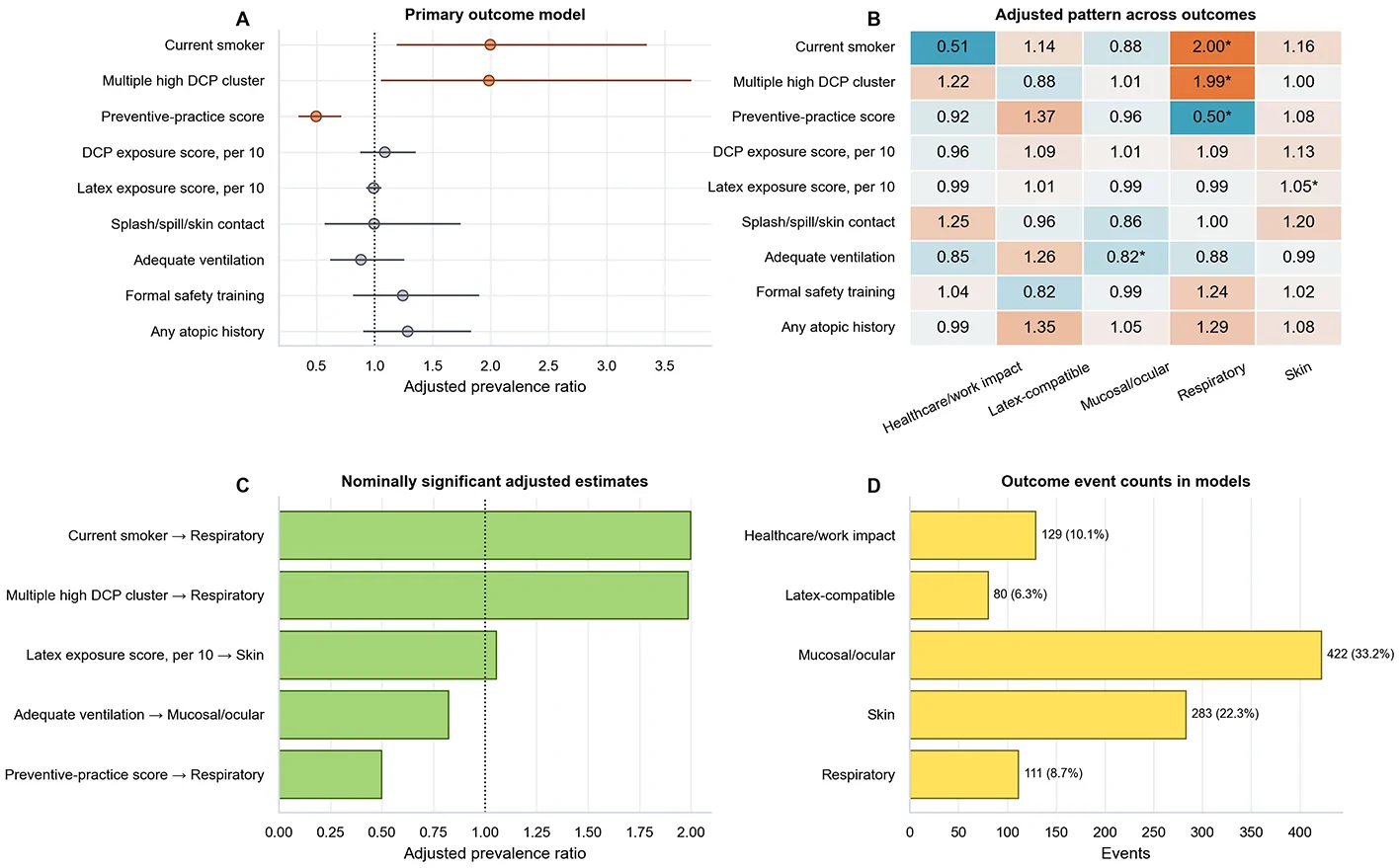
Adjusted association structure across symptom outcomes. **(A)** Forest plot of adjusted prevalence ratios (aPRs) with 95% confidence intervals for the primary outcome, work-related respiratory symptoms. **(B)** Heatmap of aPRs for all predictors across the five symptom outcomes; cell shading reflects the direction and magnitude of association, and asterisks denote nominal statistical significance (*p* < 0.05). **(C)** Nominally significant adjusted associations ranked by effect size, with the null value (aPR = 1.0) indicated. **(D)** Event counts and prevalence for each outcome included in the regression models. All estimates derived from modified Poisson regression with robust variance, adjusting simultaneously for all covariates. *N* = 1,271.

The adjusted pattern differed across outcome domains (Figure 4B). For skin symptoms, latex exposure score showed a small but significant dose effect (aPR 1.05 per 10 points, 95% CI 1.01–1.10; *p* = 0.014), and the disinfectant/sterilant score approached significance (aPR 1.13 per 10 points; *p* = 0.054). For mucosal/ocular symptoms, adequate ventilation was protective (aPR 0.82, 95% CI 0.70–0.96; *p* = 0.016), as was former smoking (aPR 0.43, 95% CI 0.23–0.81; *p* = 0.010). Educational qualification influenced the less common outcomes: relative to a bachelor's degree, diploma or other qualification was associated with higher latex-compatible symptoms (aPR 1.70; *p* = 0.031) and greater healthcare or work impact (aPR 1.54; *p* = 0.024), while a master's degree was associated with higher mucosal/ocular symptoms (aPR 1.32; *p* = 0.042). This domain-specific divergence—behavior driving respiratory risk, latex dose driving skin risk, and ventilation driving mucosal risk—is itself an important result, indicating that no single intervention will address the full symptom spectrum and that prevention must be matched to the target outcome. The nominally significant associations are summarized in Figure 4C, and outcome event counts confirmed adequate statistical power for the more common outcomes (mucosal/ocular *n* = 422; skin *n* = 283) relative to the rarer ones (latex-compatible *n* = 80) (Figure 4D).

### 3.6 Awareness, preventive practice, and barriers

Factual safety awareness was uneven across the eight knowledge items (Figure 5B). Overall, 644 of 1,271 nurses (50.7%) met the *a priori* threshold for adequate safety awareness (total score ≥6 of 8 items; Section 2.7), consistent with the summary figure reported in the Abstract. Recognition was highest for the general irritant hazard of disinfectants (82.1% correct) but fell below 60% for the limitations of surgical masks during spill cleanup (57.1%) and the non-universal protection offered by latex gloves (58.5%), marking these as the principal awareness gaps. The priority matrix, which plots correct-response proportion against the unadjusted respiratory prevalence ratio, identified awareness and practice items clustering below the 60% favorable-response threshold as the highest-priority intervention targets (Figure 5A). By ranking items simultaneously on prevalence and knowledge deficit, this matrix converts descriptive data into a concrete training agenda—a translational step rarely provided by comparable surveys.

**Figure 5:**
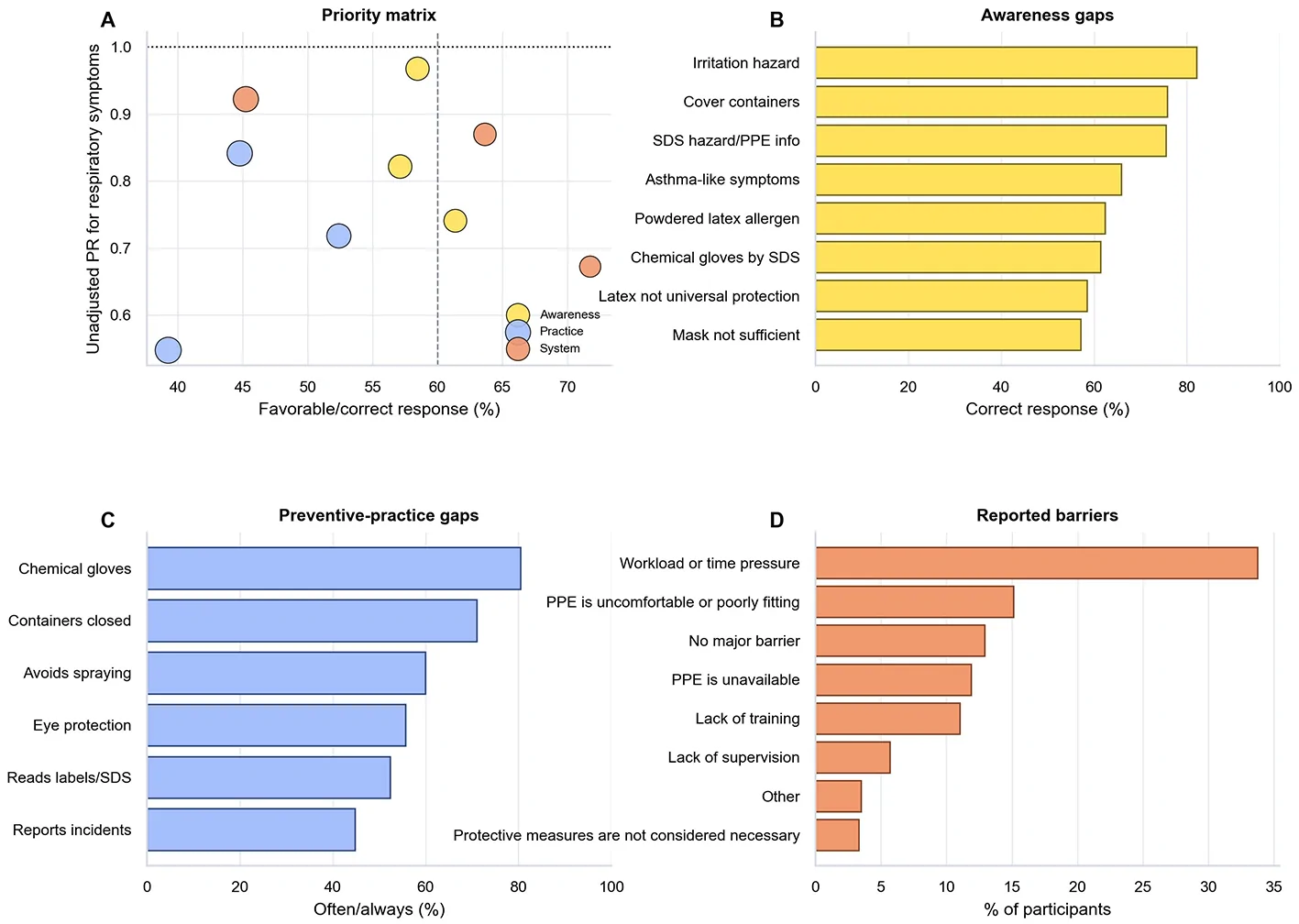
Awareness, prevention, and implementation priorities. **(A)** Priority matrix plotting the favorable or correct-response proportion (x-axis) against the unadjusted respiratory-symptom prevalence ratio (y-axis) for awareness (yellow), practice (blue), and system (orange) items; items below the favorable-response threshold represent priority intervention targets. **(B)** Correct-response proportions for the eight occupational-safety awareness items, ranked from highest to lowest. **(C)** Preventive-practice items reported as performed often or always, ranked by prevalence. **(D)** Self-reported main barriers to consistent use of protective measures, expressed as the percentage of participants. *N* = 1,271.

Preventive practice was similarly inconsistent (Figure 5C). Although chemical-appropriate glove use was relatively high (80.5% often/always), incident reporting (44.8%), label or SDS review (52.4%), and eye protection during splash-risk tasks (55.6%) were the weakest behaviors. The dominant self-reported barrier to consistent protection was workload or time pressure (33.8%), substantially exceeding PPE discomfort or poor fit (15.1%) and PPE unavailability (11.9%) (Figure 5D). That the leading barrier is organizational rather than material indicates that supplying equipment alone will not close the practice gap without addressing staffing and workflow.

### 3.7 Safety infrastructure and symptom burden

The Safety Infrastructure Index (SII) was distributed similarly across OR roles, with no significant between-role difference (Kruskal–Wallis *p* = 0.862) (Figure 6A), and ordered logistic regression identified no individual determinant of higher SII (Figure 6B). Mucosal/ocular symptom prevalence declined gradually with rising SII. Skin symptom prevalence dropped sharply between the lowest SII level and the next level before plateauing at higher SII levels, rather than declining gradually across the full range. The cumulative symptom-count trend did not reach significance (*p* = 0.065) (Figure 6C). Path analysis showed that safety infrastructure was strongly associated with stronger preventive practice (β = 0.36; *p* < 0.001), but its indirect effect on overall symptom burden through practice was negligible and non-significant (indirect β = −0.01, 95% CI −0.03 to 0.02) (Figure 6D). This dissociation is informative rather than null: institutional infrastructure shapes how nurses behave but does not, on its own, translate into lower aggregate symptom burden, implying that infrastructure and individual behavioral reinforcement must act together.

**Figure 6:**
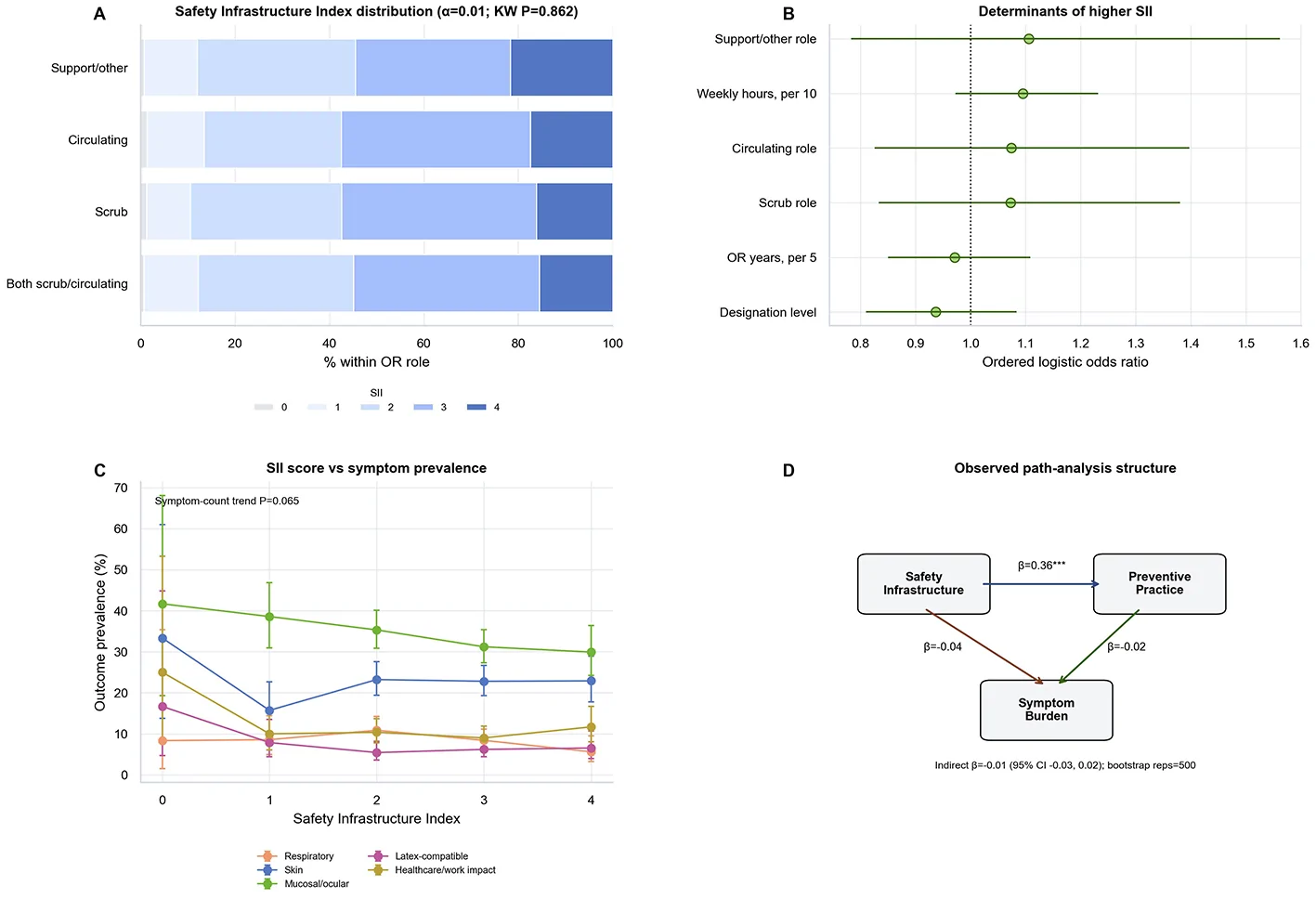
Safety infrastructure adequacy and symptom burden. **(A)** Distribution of the Safety Infrastructure Index (SII, range 0–4) within each OR role, with internal consistency (α) and the Kruskal–Wallis test for between-role difference reported. **(B)** Ordered logistic regression of determinants of higher SII, shown as odds ratios with 95% confidence intervals. **(C)** Prevalence of each symptom outcome across SII levels, with the trend test for cumulative symptom count reported; error bars show 95% confidence intervals. **(D)** Observed path-analysis structure relating safety infrastructure, preventive practice, and symptom burden, with standardized path coefficients and the bootstrapped indirect effect (95% CI) reported. *N* = 1,271.

## 4 Discussion

In this single-center study of 1,271 operating-room (OR) nurses in China, occupational chemical exposure was widespread but unevenly distributed across agents, and work-related symptoms were common, with mucosal or ocular complaints (33.2%) and skin symptoms (22.3%) substantially exceeding respiratory symptoms (8.7%). Three findings carry the greatest weight. First, the agents responsible for the bulk of regular exposure were not the classic high-level sterilant but the everyday antiseptics and surface disinfectants. Second, in adjusted models the respiratory symptom burden was associated with concurrent multi-agent exposure and current smoking, yet a substantially lower prevalence was associated with individual preventive practice. Third, institutional safety infrastructure shaped preventive behavior but did not, on its own, reduce aggregate symptom burden. Together these results map a differentiated risk architecture that has not previously been described in a single OR nursing cohort.

The exposure gradient we observed inverts the conventional regulatory hierarchy. Alcohol, chlorhexidine and povidone-iodine antiseptics (54.1% regular exposure), latex products (42.7%), quaternary ammonium compounds (QACs, 38.6%), and chlorine (32.5%) dominated, while glutaraldehyde (23.6%), ethylene oxide (10.7%), and peracetic acid (9.7%) ranked lowest. This matters because surveillance and engineering controls in many institutions remain oriented toward aldehyde sterilants, whose airborne concentrations historically prompted exposure-limit legislation (7). Our data indicate that the modern OR exposure burden has migrated toward frequently used, low-perceived-risk agents—a pattern consistent with the French CONSTANCES cohort, in which QAC-cluster nurses showed elevated asthma symptom scores (11), and with US. data linking surface cleaning, QACs, bleach, and sprays, rather than instrument sterilants, to new-onset asthma (10). The unadjusted agent-level analysis reinforces this: glutaraldehyde (PR 1.49) and formaldehyde (PR 1.54) were the only agents individually associated with respiratory symptoms (Table 1), but their low population exposure frequency limits their attributable contribution relative to the high-frequency antiseptics.

The most prevalent outcome was mucosal/ocular symptoms, the domain least often measured in occupational studies of healthcare workers, which have overwhelmingly prioritized asthma or hand dermatitis in isolation (7, 10). By assessing four symptom domains in one cohort, we found that respiratory symptoms—the conventional focus—were in fact the least common of the chemically attributable outcomes. This does not contradict the asthma literature so much as reframe it: respiratory endpoints capture only a fraction of the total chemically related morbidity, and a respiratory-only design would have missed roughly four-fifths of the symptom burden in this population. The protective association of adequate ventilation with mucosal/ocular symptoms specifically (aPR 0.82; *p* = 0.016), but not with respiratory or skin outcomes (Table 4, Figure 4B), is mechanistically coherent: airborne irritant vapor concentration is the proximate driver of conjunctival and upper-airway irritation, and is the exposure most directly modified by local exhaust and dilution ventilation.

Current smoking associated with a doubling of respiratory symptom prevalence (aPR 2.00; *p* = 0.009), and membership in the multiple high disinfectant/cleaning-product (DCP) exposure cluster conferred a nearly identical increase (aPR 1.99; *p* = 0.033). The smoking association is biologically plausible: tobacco smoke and chemical irritants share oxidative and mucin-hypersecretory airway pathways, and current smoking is a recognized amplifier of airway mucin production and symptom expression (27). The co-exposure finding supports treating simultaneous multi-agent contact, rather than any single chemical, as the relevant unit of respiratory risk—an approach validated externally by the CONSTANCES cluster analysis (11) but rarely operationalized in single-center surveys.

The strongest single association in our analysis was protective: each one-point rise in the preventive-practice score was associated with approximately half the respiratory symptom prevalence (aPR 0.50; *p* < 0.001). This is the central translational message. Although exposure is structurally determined, the respiratory consequence is substantially behaviorally modifiable. The finding aligns with evidence that knowledge and awareness predict protective-equipment compliance among nurses (24), and the moderate awareness–practice correlation we observed (ρ = 0.55; Figure 3D) is consistent with that pathway.

An apparent paradox warrants comment. Formal safety training was not protective for any outcome and trended toward higher respiratory prevalence (aPR 1.24; Table 4). This is most plausibly reverse causation: nurses in higher-exposure roles, or those already symptomatic, are preferentially selected into institutional training, inflating the crude training–symptom association in a cross-sectional frame. A similar discordance between training receipt and outcome has been noted where training is reactive rather than preventive (24, 28, 29). The contrast between ineffective training receipt and effective enacted practice suggests that training content is not translating into the specific behaviors—eye protection, incident reporting, label review—that were weakest in our cohort (Figure 5C).

Latex products were the second most frequent regular exposure, and the latex exposure score showed a small but significant dose relationship with skin symptoms (aPR 1.05 per 10 points; *p* = 0.014), while latex-compatible immediate symptoms affected 6.3% of nurses. This sits within a literature describing substantial reductions in latex allergy following powder-free and non-latex substitution in high-income settings (16). UK surveillance data documented a significant post-intervention fall in latex-associated allergic contact dermatitis among healthcare workers, alongside a coincidental rise in irritant contact dermatitis (30). Our data are consistent with a transitional state: powdered latex still reached 15.2% regular exposure (Figure 1B), indicating that the substitution that has nearly eliminated the problem elsewhere remains incomplete in this setting, and that latex-compatible symptom surveillance retains relevance here even where it has waned in other regions.

The dissociation in our path analysis is one of the study's more instructive results. Safety infrastructure was strongly associated with stronger preventive practice (β = 0.36; *p* < 0.001), but its indirect effect on overall symptom burden through practice was negligible (indirect β = −0.01; 95% CI −0.03 to 0.02, Figure 6D), and the Safety Infrastructure Index did not differ across OR roles (Kruskal–Wallis *p* = 0.862). Two explanations are likely. First, infrastructure availability and infrastructure use diverge: workload or time pressure was the dominant barrier to protection (33.8%; Figure 5D), far exceeding equipment unavailability (11.9%), so resources present on paper may not be used at the point of exposure. Second, aggregating five heterogeneous outcomes into a single symptom-burden score dilutes domain-specific effects—ventilation's genuine protective effect on mucosal symptoms is averaged against its null effect on skin and respiratory outcomes (31, 32). The policy implication is direct: supplying equipment without relieving the organizational pressures that prevent its use is unlikely to lower symptom burden, echoing the conclusion that barriers to protection in nursing are predominantly organizational rather than material (24, 32, 33, 48).

Although the aggregate exposure burden was dominated by antiseptics and surface agents, the only agents individually associated with respiratory symptoms in unadjusted analysis were glutaraldehyde and formaldehyde (Table 1). This dissociation between population exposure frequency and per-agent hazard is mechanistically important and concordant with specialist surveillance data. In a UK occupational lung disease service, the cleaning agents most frequently identified as causing occupational asthma over 16 years were chloramines, glutaraldehyde, and quaternary ammonium compounds (QACs), confirming that aldehydes retain disproportionate sensitizing potency even as their use declines (34, 35). Our finding that QACs reached 38.6% regular exposure yet showed no individual respiratory association (PR 0.97) does not exonerate them; QACs are established sensitizers capable of inducing both irritant and IgE-mediated airway responses (15, 36), and their apparent neutrality here more likely reflects the cross-sectional design and the dilution of any single high-frequency agent's effect within the multi-agent cluster. The practical inference is that exposure-frequency ranking alone is an unreliable guide to hazard prioritization; agent-specific toxicological potency must be weighed alongside population exposure when designing OR substitution programs (37, 38).

The high mucosal/ocular prevalence we observed should be interpreted within the broader multi-hazard reality of the OR, where chemical agents coexist with surgical smoke and aerosolized particulates. A cross-sectional hospital study in Turkey found that operating rooms ranked among the riskiest units for work-related health complaints and that sterilization and disinfection agents, formaldehyde, and surgical smoke were each linked to respiratory and dermal complaints (39). Surgical smoke specifically is an increasingly recognized OR airway hazard that our instrument did not capture, and which plausibly contributes to the mucosal and ocular symptom load attributed here to chemical agents (40). This represents both a limitation and a direction for future work: disentangling the chemical and combustion-product contributions to upper-airway and ocular symptoms will require instruments that jointly assess disinfectant exposure and smoke exposure. The protective effect of ventilation we identified for mucosal outcomes (aPR 0.82) is consistent across both hazard classes, since local exhaust and effective air exchange reduce airborne concentrations of vapors and smoke alike, strengthening the case for ventilation as a shared engineering control (12, 40).

Among the weakest preventive behaviors in our cohort was incident reporting (44.8% often/always; Figure 5C), and a substantial minority of nurses were unfamiliar with the institutional reporting pathway, though this was not captured in the composite awareness score. This reporting deficit has direct consequences for the validity of occupational surveillance and for the interpretation of our own prevalence estimates. Under-reporting of occupational incidents is well documented among healthcare workers; in a study of junior doctors, 31% of those sustaining needlestick injuries did not report them, with perceived low risk and lack of time as the dominant reasons (40, 41) — the same organizational pressures our nurses cited as barriers to protection. If symptom-triggering exposure events are systematically under-reported, institutional incident registries will understate true burden, and passive surveillance will fail to detect the high-frequency, low-acuity chemical exposures that our active questionnaire revealed. This argues for periodic active symptom surveillance, rather than reliance on spontaneous reporting, as the appropriate monitoring strategy for OR chemical hazards, and frames our cross-sectional instrument as a prototype for such surveillance (42–44).

The relevance of these findings extends beyond the index hospital. Although the absolute exposure and symptom prevalences should not be assumed to generalize directly from a single tertiary center, the integrated assessment of agent-specific exposure, preventive behavior, engineering controls, workload, and multi-domain symptoms provides a framework that may facilitate comparative occupational-health evaluation across perioperative settings internationally. Occupational health surveillance infrastructure for healthcare workers remains underdeveloped across many low- and middle-income settings, where competing priorities and workforce shortages constrain preventive programs (8, 44, 49–51). In China specifically, occupational exposure research among healthcare workers has concentrated on needlestick and sharps injuries (17) and on workforce stress under staffing pressure (20), while chemical disinfectant exposure has received comparatively little attention. Our data fill part of that gap by demonstrating that the chemical exposure burden in a Chinese tertiary OR is substantial, multi-domain, and behaviorally modifiable. The dominance of workload as the principal barrier to protection (33.8%) is particularly salient in a system characterized by high nurse-to-patient ratios and chronic time pressure (20), and aligns with evidence that excessive workload leads healthcare workers to delay or omit PPE use (39). Interventions transplanted from high-income settings—where latex substitution and aldehyde phase-out have largely succeeded—cannot be assumed to translate without adaptation to the staffing and infrastructure realities of this context (45, 46).

Three implications follow directly from the differentiated risk architecture we describe. First, because preventive practice halved respiratory risk while training receipt did not, occupational health programs should shift emphasis from didactic training completion toward measurable enactment of specific behaviors—particularly the three weakest in our cohort: eye protection, label/SDS review, and incident reporting. Behavior-specific competency assessment, rather than attendance-based training metrics, is the appropriate quality indicator. Second, because the dominant barrier was organizational, equipment provision must be paired with workflow and staffing measures that make protected practice feasible during high-pressure procedures; evidence consistently links nurse workload and staffing to safety-process adherence and patient outcomes (13, 47). Third, the agent-specific findings support a targeted substitution and ventilation strategy: retaining vigilance for aldehyde sensitizers while recognizing that the high-frequency antiseptics and QACs driving population exposure warrant their own engineering controls and surveillance. The priority matrix (Figure 5A) operationalizes this by ranking intervention targets on the joint axes of prevalence and knowledge deficit, providing a reproducible template that other institutions can apply to their own surveillance data.

Several limitations effect interpretation. The cross-sectional design precludes causal inference and is vulnerable to the healthy worker effect, whereby symptomatic nurses leave OR work before assessment, biasing exposure–outcome associations toward the null (9); this likely explains, in part, the modest exposure-score associations and is consistent with the null disinfectant–asthma findings of a large prospective nursing cohort attributed to the same mechanism. All outcomes were self-reported symptoms, not clinically confirmed diagnoses, and are subject to recall and reporting bias; we deliberately framed them as surveillance endpoints rather than diagnoses of asthma, contact dermatitis, or latex allergy. Single-center sampling limits generalizability beyond comparable Chinese tertiary hospitals, though the census approach minimizes selection bias within the source population. Data collection was conducted within a defined three-month window (February–April 2026), during which institutional staffing, infection-control policy, product formulary, and ventilation practices were stable; all observations therefore originated from a single contemporaneous calendar period rather than from data collected across multiple years, minimizing the potential for secular period effects. However, a 63% response rate does not eliminate the possibility of selection or non-response bias; nurses who declined may differ systematically in exposure or symptom status from those who responded, and the direction of any resulting bias cannot be determined from the available data.

## 5 Conclusion

Among operating-room nurses, work-related symptoms were common and dominated by mucosal, ocular, and skin complaints rather than respiratory ones, while the bulk of regular chemical exposure arose from everyday antiseptics and surface disinfectants rather than classic high-level sterilant. Respiratory risk was concentrated among nurses with concurrent multi-agent exposure and current smoking, yet a substantially lower prevalence was associated with consistent preventive practice, whereas environmental controls were associated selectively with a lower prevalence of mucosal and ocular symptoms. Institutional infrastructure shaped protective behavior but did not, by itself, lower overall symptom burden, and workload rather than equipment availability was the principal barrier to protection. These findings indicate that effective prevention must pair behavior-specific competency, targeted substitution, and ventilation controls with organizational measures that make protected practice feasible during high-pressure surgical work. Active symptom surveillance, in place of reliance on spontaneous reporting, is warranted to monitor this modifiable occupational burden.

## Data Availability

The raw data supporting the conclusions of this article will be made available by the authors, without undue reservation.
